# The biodiscovery potential of marine bacteria: an investigation of phylogeny and function

**DOI:** 10.1111/1751-7915.12054

**Published:** 2013-04-04

**Authors:** Martin Mühling, Ian Joint, Andrew J Willetts

**Affiliations:** 1 TU Bergakademie Freiberg, Institute of Biological SciencesLeipziger Str. 29, 09599, Freiberg, Germany; 2 Plymouth Marine Laboratory, Prospect PlaceThe Hoe, Plymouth, PL1 3DH, UK; 3 Marine Biological Association UK, The LaboratoryCitadel Hill, Plymouth, PL1 2PB, UK; 4 Curnow ConsultanciesTrewithen House, Ashton, Helston, TR13 9PQ, UK

## Abstract

A collection of marine bacteria isolated from a temperate coastal zone has been screened in a programme of biodiscovery. A total of 34 enzymes with biotechnological potential were screened in 374 isolates of marine bacteria. Only two enzymes were found in all isolates while the majority of enzyme activities were present in a smaller proportion of the isolates. A cluster analysis demonstrated no significant correlation between taxonomy and enzyme function. However, there was evidence of co-occurrence of some enzyme activity in the same isolate. In this study marine *Proteobacteria* had a higher complement of enzymes with biodiscovery potential than *Actinobacteria*; this contrasts with the terrestrial environment where the *Actinobacteria* phylum is a proven source of enzymes with important industrial applications. In addition, a number of novel enzyme functions were more abundant in this marine culture collection than would be expected on the basis of knowledge from terrestrial bacteria. There is a strong case for future investigation of marine bacteria as a source for biodiscovery.

## Introduction

In the last decade, molecular biology techniques have been widely applied to marine and terrestrial microbial assemblages, and have led to a revolution in understanding of natural diversity. With that new understanding came the suggestion that genomic and metagenomic data would open up new opportunities to exploit genetic information from natural assemblages for biodiscovery (Heidelberg *et al*., 2010). However, that promise is largely unfulfilled and the isolation of microbial cultures remains the best approach to date to develop novel processes and to utilize novel enzymes. For the immediate future, it may be that the best use of environmental genomic data will be to indicate those biochemical pathways that exist in microbes with no representative isolates presently in laboratory culture.

There is no doubt that understanding of natural assemblages is progressing rapidly and that this knowledge should benefit biodiscovery research. Whereas 25 years ago we knew little more than that there were about ∼10^6^ bacteria in each millilitre of sea water, but with no understanding of how many species might exist, we now know that microbial diversity is huge. For example, in a 6-year study of bacterial diversity in the English Channel, Gilbert and colleagues (2012) have shown that there are more than 20 000 distinct bacterial operational taxonomic units (OTUs – strictly 16S rRNA genes). In addition to describing overall microbial diversity, modern sequencing technologies are accessing metagenomes (Venter *et al*., 2004; Tringe *et al*., 2005; DeLong *et al*., 2006; Dinsdale *et al*., 2008) and transcriptomes (Moran *et al*., 2007; Gilbert *et al*., 2008) of microbial assemblages in the natural environment. These studies now open up the possibility to compare genetic information and gene expression in different microbial assemblages, thus forming a basis for the study of the overall activity and function of the microbial population in the environments under investigation.

In terms of developing new biotechnological products, these novel approaches should be identifying a number of functional genes and enzymes with a high potential for industrial and/or pharmaceutical applications (for reviews see Streit *et al*., 2004; Steele *et al*., 2009). Yet few practical applications of this metagenomic knowledge have resulted to date. A number of limiting factors may be involved – mostly linked to problems in functional screening of potentially useful genes. First, the genes of interest must be cloned and expressed in a heterologous host (usually *Escherichia coli*) which may introduce associated problems concerned with, among other things, appropriate promoters, regulators, relevant cofactors. Additionally, expression must result in sufficient levels of enzymatic activity to be detected in biochemical assays. Finally, heterologously expressed proteins may prove to be toxic to the host bacterium and thus result in culture death.

To date, the most successful approach remains the isolation from the environment of new cultures, with enzymes and activities suitable for biotechnology. Most of the effort to isolate bacteria for biodiscovery has focused on heterotrophic bacteria from the terrestrial environment. Particular emphasis has been on members of the phylum *Actinobacteria* (Bull *et al*., 2000). These bacteria are excellent candidates because they have large genomes with an extensive biochemical complement and are well-established producers of a number of enzymes of current industrial importance (Faber, 2004; Suneetha and Khan, 2011).

However, over the last decade, as more metagenomic data have become available, it has been suggested that marine bacteria, including both *Actinobacteria* (Bull and Stach, 2007) and other major bacterial groups (Wagner-Döbler *et al*., 2002), possess a wide range of enzymes with novel substrate specificities and novel enzymatic activities that should make them attractive candidates for biodiscovery.

The outcomes of the relatively few studies conducted to date suggest that, while marine *Actinobacteria* do indeed appear a major source of biotechnologically relevant enzymes (Trincone, 2011), marine strains belonging to other phylogenetic phyla have also been reported to contain relevant enzymes (e.g. Trincone, 2010). However, few large-scale systematic analyses have so far been undertaken to assess the general suitability of these various marine bacterial groups.

In this article, 374 marine isolates have been screened for 34 different enzymatic activities. The selected activities used existing enzymatic assays but also targeted activities of particular biotechnological relevance; that is, there was either an established or perceived industrial demand (Kirk *et al*., 2002; Tang and Zhao, 2009), as well as a good level of scientific understanding, with a significant probability of progress leading to biotechnological advance. Priorities were to test if specific phylogenetic groups of bacteria were more likely to harbour particular sets of relevant enzymatic activities – that is, to test if biochemical function might be linked to broad phylogenetic groups of marine bacteria. This is unlikely since, (with some exceptions such as the clades of bacteria and archaea responsible for ammonia and nitrite oxidation; see for example Kowalchuk and Stephen, 2001) phylogeny does not usually correlate to metabolic function; nevertheless it is a valid hypothesis to test. All of the isolates were identified to the level of genus, so allowing the application of multivariate statistical methods to identify possible links between the phylogenetic identity of an isolate and enzyme activities. Since previous enzyme-screening studies have focused on terrestrial microorganisms, a second objective was to investigate if the distribution of enzymatic capabilities among bacteria from the marine environments is intrinsically different from that known for the terrestrial environment – which could shed new insights into whether or not fundamentally different environments have shaped microbial evolution and diversity in the sea which, in turn, would provide greater potential for biodiscovery.

## Results

### Screening for enzymatic activities

There was large variation in the distribution of the various enzymatic activities among the 374 bacterial isolates (Table 1). Two enzyme activities, C4- and C16-carboxy esterases, were detected in all 374 isolates; these activities have been categorized as ‘core’ enzymes and were indeed expected to occur in the vast majority of isolates. Other ‘core’ activities were also detected in many of the isolates. Both EC1.1-type and EC1.3-type dehydrogenases were commonly found (in more than 344 isolates) but other ‘core’ enzymes were detected less frequently. Indeed, one enzyme that was assumed would have a ‘core’ metabolic function, acid phosphomonoesterase, was only found in one isolate (16S rRNA-based sequence comparison indicates the highest similarity of this isolate to uncultured members of the genus *Pseudomonas*). As was anticipated, the majority of enzymes were found in a small proportion of the isolates.

**Table 1:** Distribution of enzymes among the 374 bacterial isolates screened in this study, and the characteristic of the individual enzyme based on its biological role (i.e. ‘core’ metabolism or ‘specialist’ function)

Enzyme	Substrate^a^	Occurring in strains^b^	Role	Enzyme code No.
C4-carboxy esterase		374	Core	9
C16-carboxy esterase		374	Core	10
Peroxidase		360	Specialist	14
Laccase		355	Specialist	15
EC1.3-type dehydrogenase		351	Core	13
EC1.1-type dehydrogenase	Isopropyl alcohol	345	Core	11
EC1.1-type dehydrogenase	dl-threonine	343	Core	12
Alkaline phosphodiesterase		244	Core	8
Alkaline phosphomonoesterase		220	Core	6
β-Halocarboxylic acid dehalogenase		209	Specialist	30
Epoxyalkene hydrolase		188	Specialist	17
α-Halocarboxylic acid dehalogenase		134	Specialist	29
γ-Halocarboxylic acid dehalogenase		133	Specialist	31
Acid phosphodiesterase		112	Core	7
Benzoic acid-induced monooxygenase		81	Specialist	20
m-Toluic acid induced monooxygenase		58	Specialist	21
Indole-induced monooxygenase		57	Specialist	19
1, 2-Dione reductase	2,3-Butanedione	51	Specialist	18
m-Halobenzoic acid dehalogenase		46	Specialist	33
Nitrile hydratase (aliphatic)	Propionitrile	34	Specialist	4
Epoxystyrene hydrolase		32	Specialist	16
Nitrile hydratase (aromatic)	Benzonitrile	30	Specialist	3
Nitrilase (aromatic)	Benzonitrile	27	Specialist	1
Nitrilase (aliphatic)	Propionitrile	25	Specialist	2
m-Toluic acid induced dioxygenase		25	Specialist	24
o-Halobenzoic acid dehalogenase		19	Specialist	32
p-Halobenzoic acid dehalogenase		17	Specialist	34
Benzoic acid-induced dioxygenase		10	Specialist	23
3-Acetylindole-induced BVMO		8	Specialist	25
Lactone hydrolase		8	Specialist	28
Cyclohexanone-induced BVMO		6	Specialist	26
Acetophenone-induced BVMO		4	Specialist	27
Indole-induced dioxygenase		4	Specialist	22
Acid phosphomonoesterase		1	Core	5

a Details on enzymatic assays are provided in Table S1.

b Out of a total of 374 strains.

The enzyme code numbers in the final column are those used to identify the enzyme functions in Fig. 3.

**Figure 1:**
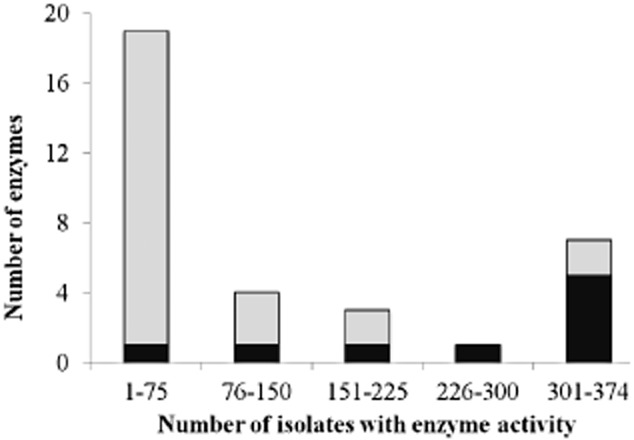
Frequency of the occurrence of the 34 enzyme activities tested among the 374 isolates screened in the study. Black and grey bars indicate ‘core’ and ‘specialist’ enzymes respectively (see Table 1).

Figure 1 illustrates the frequency distribution of enzyme activity among the 374 isolates. The results have been binned into 20% fractions of the total 374. Seven enzyme activities were most widely detected and occurred in between 300 and 374 isolates. Of these seven enzymes, five were designated as ‘core’ and two were ‘specialist’. At the other end of the frequency distribution, 19 enzymes were found in the lower 20% of the distribution spectrum, and were detected in 75 isolates or fewer. As mentioned above, this included acid phosphomonoesterase, which was assumed to be a ‘core’ enzyme – but which was only detected once. The remaining ‘core’ enzyme activities were detected more frequently, but did not occur in as many isolates as had been expected. For example, acid phosphodiesterase was detected in 112 isolates (Table 1, Fig. 1) and alkaline phosphomonoesterase and alkaline phosphodiesterase in 220 and 244 isolates respectively. Two ‘specialist’ enzyme activities were very widely distributed: peroxidase and laccase were detected in 361 and 356 isolates respectively.

### Evaluating potential links between taxonomy and enzyme activity

Cluster analysis was used to assess if specific bacterial groups possess particular enzymatic activities. The first approach compared enzymatic activity and the taxonomic identity of each of the 374 isolates at various taxonomic levels (phylum, class, genus – see Table S1). This matrix was then evaluated by calculating the similarities between every pair of isolates using the simple-matching coefficient. Subsequent hierarchical agglomerative clustering with group-average linkage of the resulting similarity matrix revealed that the 374 isolates were grouped into 18 significant clusters of which three consisted of only one or two isolates (results not shown). This analysis did not reveal any obvious pattern or correlation between taxonomy and function. That is, there were no significant correlations between taxonomic group (at any level from genus to phylum) of the isolates and the enzymatic functions that they possess. Similar results were obtained when the same analyses were carried out using a reduced data set consisting only of the presence/absence data for the specialist enzymes.

**Figure 2:**
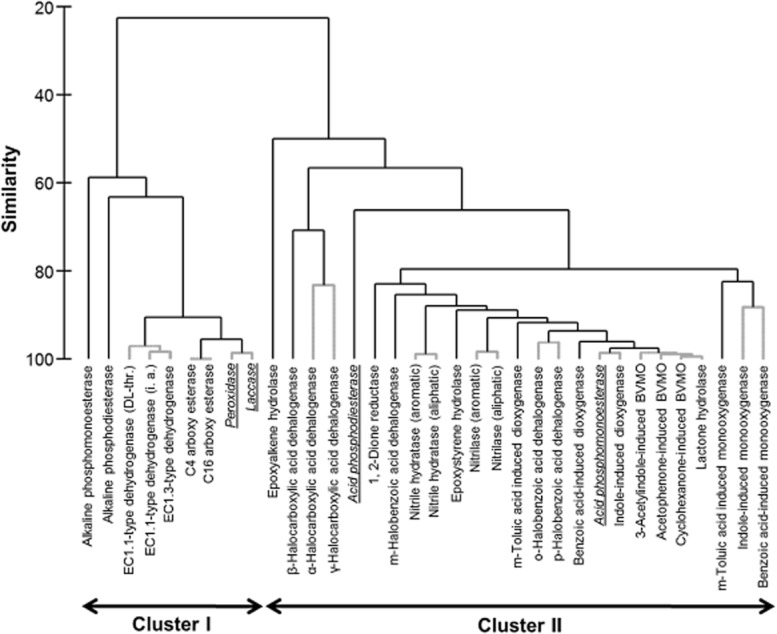
Dendrogram showing the results from the cluster analysis of the co-occurrence of enzymatic activities among the 374 bacterial isolates. In the case of the EC1.1-type dehydrogenase activities, the abbreviations in parentheses indicate dl-threonine (DL-thr.) and isopropyl alcohol (i. a.). Grey dotted lines indicate where the distinction of enzymatic activities into different clusters could have arisen by chance (SIMPROF test). The 34 enzymes are divided into two main clusters (I, II), which closely resembled the definition of ‘core’ (cluster I) and ‘specialist’ (cluster II) enzymes. Enzymes not meeting this definition are indicated in italics and are underlined.

The data were also interrogated for potential co-occurrence of particular enzymatic activities among the 374 isolates. This represents a test for the possibility that the presence of one enzyme activity could be used as a proxy for the presence of another enzyme. Applying the same statistical approach used for the previous analysis revealed two distinct clusters – enzyme cluster I and II (Fig. 2). Enzyme cluster I consisted of nine enzymes, of which seven could be regarded as ‘core’ enzymes (Table 1), while those that are grouped into enzyme cluster II were predominantly (23 out of 25 enzymes) ‘specialist’ enzymes (Table 1). This clustering suggests the co-occurrence of core enzymes or of specialist enzymes among particular isolates.

The significance of the subclustering into the two enzyme clusters was further evaluated using the SIMPROF test for multivariate structure. The results from this analysis supported the division of the enzymes into the two main clusters at a level of approximately 22% similarity, and also provide further evidence of significant multivariate structure even below this division (Fig. 2).

### Enzyme activity associated with major taxonomic groups

**Figure 3:**
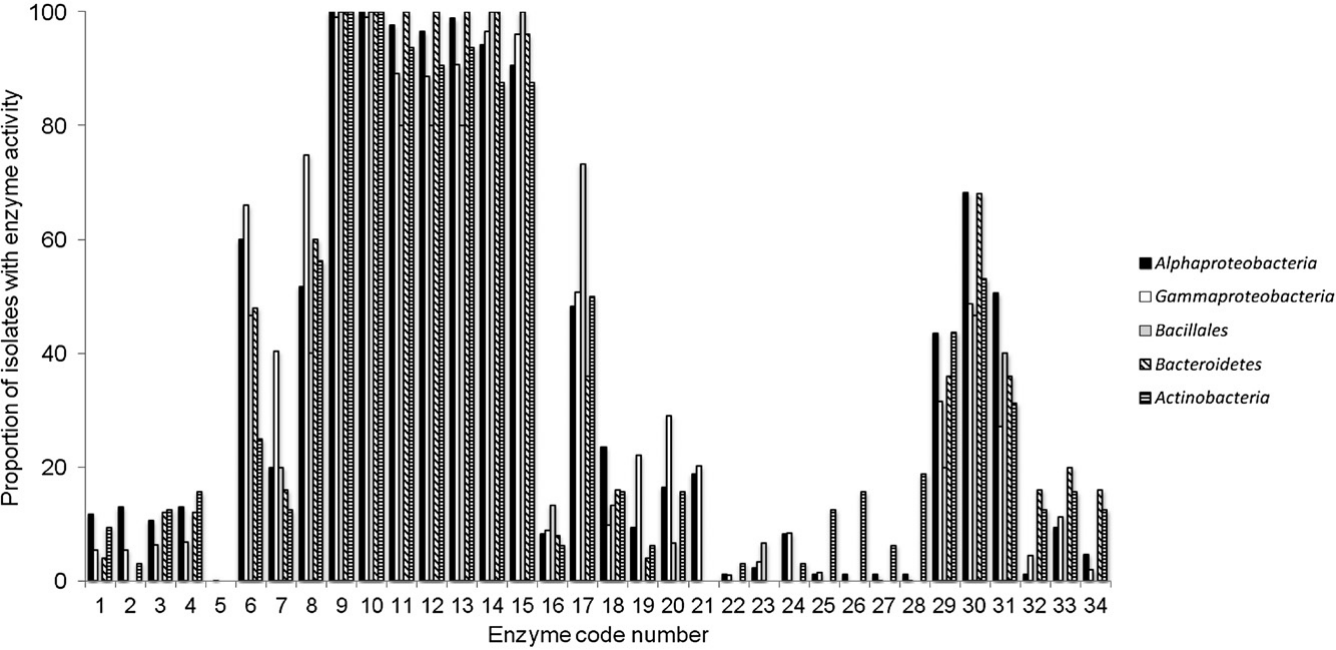
Taxonomic relationship with enzyme function. Enzyme activity has been allocated to the major bacterial groupings of *Alphaproteobacteria*, *Gammaproteobacteria*, *Bacillales*, *Bacteroidetes* and *Actinobacteria*. The *y*-axis shows the proportion of isolates in each group that possess a specific enzyme activity. Enzymes are identified by code number (Table 1).

Based on their 16S rRNA gene fragment sequence 374 isolates were identified as members of the following five phyla of bacteria: *Proteobacteria* (with 88 in the class *Alphaproteobacteria*, two in the class *Betaproteobacteria* and 203 in the class *Gammaproteobacteria*), *Firmicutes* (15 – all of which belonged to three genera: 13 to *Bacillus*, one to *Geobacillus* and one to *Planococcus*), *Bacteroidetes* (CFB) group (32), *Actinobacteria* (33) and *Verrucomicrobia* (one). Figure 3 shows the proportion of each group of isolates that possessed each of the tested enzyme activities. Seven enzyme activities were detected in approximately 90% or more of the isolates in each taxonomic group. As would be expected from Table 1, these were C4-carboxyesterase, C16-carboxyesterase, EC1.1-type dehydrogenase [substrate isopropyl alcohol], EC1.1-type dehydrogenase [substrate dl-threonine], EC1.3-type dehydrogenase, peroxidase and laccase. There was very little difference in the distribution of these enzyme activities between the five most abundant taxonomic groups. However, there were differences in the distribution of other activities. Alkaline phosphomonoesterase (which we had designated a ‘core’ enzyme) was detected in > 60% of the *Alphaproteobacteria* and *Gammaproteobacteria* but was present in only *c*. 25% of the *Actinobacteria*. Of the ‘specialist’ enzymes, some were detected across the bacterial groups. For example, the three dehalogenase enzymes were present in 30–60% of the isolates from all groups, although there were differences in the distribution between the α-, β- and γ-halocarboxylic acid dehalogenases in the *Alphaproteobacteria* and *Bacteroidetes* groups (Fig. 3).

## Discussion

### Occurrence of enzymatic activities among the isolates

This study has screened 374 marine bacterial isolates for 34 enzymatic activities that were considered to have potential for biotechnological development. It was anticipated that enzymes that are known to fulfil key primary metabolic activities (termed ‘core’ enzymes in this article) would be widely distributed but that those enzymes that catalyse specific functions (termed ‘specialist’ enzymes) would be detected less frequently. This categorization is consistent with extensive knowledge of equivalent enzyme activities in a wide range of organisms, including terrestrial microorganisms (Metzler, 2001; Madigan *et al*., 2008). Although some ‘core’ enzymes were detected in all of the isolates screened (C4- and C16-carboxyesterases), other ‘core’ enzymes were detected at a much lower frequency (Table 1, Fig. 1). Indeed, the enzyme activity that was detected least frequently was acid phosphomonoesterase, which had been assumed to be a ‘core’ enzyme. In contrast, two enzymes that were assumed to be of ‘specialist’ activity (peroxidase and laccase) were very widely distributed in the 374 strains of marine bacteria (Table 1). It is not unreasonable to find that a biodiscovery project, which focused on a previously poorly characterized resource, resulted in the detection of some enzymes at unexpected frequencies.

Clustering analysis of the results from the enzyme screening (Fig. 2) demonstrated that the enzymes are grouped into two main clusters that approximated to ‘core’ and ‘specialist’ definitions, with the differences that were highlighted in Table 1. The differences between the enzyme definition and the clusters are acid phosphomono- and diesterases, laccase and peroxidase (see below).

The distribution of enzyme activities in different bacterial taxa is interesting and appears to be different from the experience of terrestrial biodiscovery research. For example, *Actinobacteria* are often considered to be an important target for biodiscovery because of the large number of enzymes of current industrial importance developed from terrestrial *Actinobacteria* (Bull *et al*., 2000). But in this study of marine bacteria, the *Actinobacteria* (albeit a small proportion of the total number of isolates tested) did not express a wide range of enzyme activities. The only enzyme activities that occurred in a much higher proportion of the *Actinobacteria* isolates than other taxa were Baeyer-Villiger monooxygenase (BVMO) and lactone hydrolase activities (Fig. 3) – and then only in a small proportion (< 20%) of the *Actinobacteria* isolates. On the basis of this screening study, it would appear that marine *Alphaproteobacteria* and *Gammaproteobacteria* are just as likely to be high-priority candidates for biodiscovery research as *Actinobacteria*.

Another aspect influencing the distribution of the enzymatic activities among the 374 strains is the specific marine environment from which the isolates originated. For example, all strains that did not show either both peroxidase and laccase activity (14 strains) or laccase activity (five strains) were derived from the planktonic samples; both activities were present in all of the isolates from the rocky shore environment. So, depending on the enzyme group targeted biodiscovery programmes might be more effective if they focused on biofilm rather than planktonic bacteria.

### The putative role of ‘core’ and ‘specialist’ enzymes in marine bacteria

The category that we refer to as ‘core’ enzymes, serves one or more key roles in the central intermediary pathways of primary metabolism. Catabolic pathways generate both ATP and a suite of 11 key metabolites [central intermediary metabolites (CIMs)]; anabolic pathways exploit these various biochemical entities to promote both net biomass generation and dynamic turnover. Thus, EC 1.1-type oxidoreductases, such as malate dehydrogenase (EC 1.1.1.38), and EC 1.3.-type oxidoreductases, such as succinate dehydrogenase (EC 1.3.5.1) are integral to the tricarboxylic acid cycle (TCA cycle): carboxyesterases (lipases) are key hydrolytic enzymes in the channelling of triglycerides into the TCA cycle: phosphoesterases are key hydrolytic enzymes in the dynamic turnover of both nucleic acids and various nucleoside cofactors. These various enzyme types were ubiquitous throughout the 374 marine isolates tested (of 75 different identified genera, plus members of at least one novel putative genus: Table S1).

‘Specialist’ enzymes, on the other hand, would be expected to be more restricted in distribution. They typically catalyse biochemical reactions that are not essential for the central pathways of intermediary metabolism, but rather have evolved to promote more idiosyncratic activities. Some such activities (as in the case of nitrilases) enable more unusual nutrients (aromatic and aliphatic nitriles) to be converted into metabolites (carboxylic acids) that can then be directly accessed by core catabolic pathways thereby generating CIMs. Other examples of ‘specialist’ enzymes (as in the case of 1,2-dione reductase, an atypical EC.1.1-type oxidoreductase), serve roles associated with aspects of generating reduced organic end-products of atypical fermentative primary metabolic pathways (Metzler, 2001; Madigan *et al*., 2008).

However, in this study there were four notable exceptions to this grouping that may indicate generic differences between the well-characterized enzymes from terrestrial bacteria and those of marine bacteria. First, acid phosphomono- and diesterases were grouped together with otherwise exclusively specialist enzymes within Cluster II (Fig. 2); the categorization of acid phosphomono- and diesterases as ‘specialist’ enzymes in this cluster analysis may be a consequence of these activities rarely being detected among the screened isolates (Table 1). Enzyme functions with acidic pH optima may also be less important for bacterial life in the sea, given that seawater generally has a pH of 8.1 (but we acknowledge that bacteria can maintain cytosolic pH against a pH gradient). The importance of external pH is supported by the fact that alkaline phosphatase (phosphodi- and phosphomonoesterase) activities were widespread among the marine bacteria tested in this study (Figs 2 and 3). Other studies have also detected alkaline phosphatase activities in marine organisms (Olsen *et al*., 1991; Chen *et al*., 1996; Xiao *et al*., 2002; Plisova *et al*., 2005; Luo *et al*., 2009). The alkaline pH of the oceans may explain why alkaline phosphodiesterase and phosphomonoesterase activities group together with other ‘core’ enzymes within Cluster I.

The second and more unexpected exception to the grouping of the assayed enzymes is the widespread distribution of intracellular laccase (EC 1.10.3.2) and peroxidase (EC 1.11.1.7) activities; a high level of abundance leads to their clustering in the ‘core’ category for this collection of marine bacteria. Although serving well-recognized ‘specialist’ roles in the extracellular degradative activities of some specialized higher fungi, both enzyme types have been relatively rarely detected in terrestrial bacteria where they occur exclusively as extracellular activities (Sharma *et al*., 2007). The apparent widespread occurrence of these two enzyme types as intracellular activities in marine bacteria is currently unexplained. However, it is noteworthy that these activities occurred in all isolates from the rocky shore environment but not in all planktonic isolates.

### Properties of some marine-derived enzyme activities

The case for the biodiscovery potential of marine bacteria is based on the hypothesis that, because the habitat is so varied, marine bacteria are likely to have enzyme properties that differ from terrestrial bacteria. This can be examined by taking as a specific example the nitrile-hydrolysing activities nitrilase (N) and nitrile hydratase/amidase (NH/A).

Research prior to the 1990s on terrestrial bacteria led to the general consensus that competent microorganisms were able to express either a nitrilase specific for aromatic nitriles, or a nitrile hydratase specific for saturated aliphatic nitriles (Linton and Knowles, 1986). However, subsequent extensive investigations confirmed that *Actinobacteria* were a predominant source of both types of nitrile-hydrolysing enzymes (Faber, 2004): these are characterized by very broad substrate specificities that encompassed aromatic, heterocyclic, plus both saturated and unsaturated aliphatic nitriles (Raadt de *et al*., 1992). Some of these enzymes proved to be constitutive, whereas others were inducible, often by non-nitrile compounds such as ε-caprolactam (Nagasawa *et al*., 1990).

In this study, most of the 72 nitrile-hydrolysing activities were characterized either as a constitutive nitrilase (43%) or as a constitutive nitrile hydratase/amidase activity (50%); a minority (7%) had constitutive activities of both types of nitrile-hydrolysing enzymes (Table 2). In each of the major categories, the respective enzyme activities were able to hydrolyse both aromatic (benzonitrile) and saturated aliphatic (propionitrile) cyano-containing substrates. Only a small percentage of the relevant isolates could hydrolyse either the aromatic or the aliphatic substrate. These distribution patterns of activity explain the close correlation of both the aromatic- and the aliphatic-specific nitrilase activities and the equivalent correlation of nitrile hydratase into two clearly distinguishable subclusters of ‘specialist’ enzymes (Fig. 2).

**Table 2:** Distribution of specific nitrile-hydrolysing activities among the 72 isolates that tested positive for nitrilase and nitrile hydratase activity

N-aromatic	N-aliphatic	NH/A-aromatic	NH/A-aliphatic	
+	+	+	+	3 isolates
+	+	+	−	1 isolate
+	−	+	+	1 isolate
				5 isolates [6.94%]
+	+	−	−	29 isolates
+	−	−	−	1 isolate
−	+	−	−	1 isolate
				31 isolates [43.05%]
−	−	+	+	32 isolates
−	−	−	+	4 isolates
				36 isolates [50.00%]

### Conclusions

Marine bacteria are strong candidates for biodiscovery research. Enzyme activities characteristic of primary metabolism with good potential for biotechnology were widely distributed among major bacterial groups included in this study. *Alphaproteobacteria* and *Gammaproteobacteria* represented most of the isolates examined in this culture collection and these groups were as good as, if not better than, *Actinobacteria* as sources of relevant enzymes. Cluster analysis demonstrated that there was some evidence of co-occurrence of some enzyme activities. It was also clear that enzymes that we considered to be ‘specialist’ could be as widely distributed as enzymes that are part of ‘core’ intermediary metabolism. Novel enzyme functions, which have not been widely reported from terrestrial bacteria, were widely distributed between marine bacteria, making a strong case for further investigations of marine bacteria for biodiscovery.

## Experimental procedures

### Strains and isolation

A total of 374 strains of marine bacteria from a larger culture collection (> 900 isolates, described by Joint *et al*., 2010) were screened for enzyme activity. The strains had been isolated from a range of environments (Table S1), although the majority were derived from samples collected from the pelagic zone of the English Channel, nine miles off the southern coast of the UK [station L4 at the Western Channel Observatory (WCO): http://www.westernchannelobservatory.org.uk/]. A second significant source was Church Reef, Wembury Beach, Devon, England (50°19′N, 4°05′W), a rocky shore environment near Plymouth.

Most of the isolates were obtained using standard plating techniques on solidified media. Joint and colleagues (2010) provide details on the media and procedures used for the isolation. In essence, all of the media were based on seawater that was collected from station L4 in the English Channel, filtered through 0.2-μm-pore-size Nuclepore filters or Whatman GFF glass fibre filters and stored at room temperature in the dark until used for media preparation. Solidification of the media was achieved using agar, agarose or Noble agar. Additionally, certain specific groups of bacteria were targeted by the use of selective media, including *Actinomycetes* and *Vibrio* isolation agars (Difco). In several cases these media were supplemented with organic substrates.

The samples used for the isolation were both untreated natural seawater samples and samples that were treated to enrich for specific phylogenetic groups that are not usually obtained by standard approaches. Treatment techniques involved, for example, incubation with antibiotic or heat treatment to select for and encourage growth of members of the *Actinobacteria* – a group of bacteria that is one of the least abundant in the marine environment. In other cases the samples were incubated in a diffusion chamber with the seawater sample being separated by a 0.1-μm-pore-size polycarbonate membrane filter from a natural sample of marine phytoplankton species (see Joint *et al*., 2010 for details).

### Identification of isolates using PCR amplification and sequencing

All of the isolates were identified to the genus by sequence analysis of a fragment of the 16S rRNA gene (see below). To protect against genetic drift after isolation all strains were maintained at −80°C as a cell suspension in marine broth and 20% (v/v) glycerol. A *c*. 1.5 kb fragment of the 16S rRNA gene was amplified from each of the isolates using PCR primers 9bfm (5′-GAGTTTGATYHTGGCTCAG-3′; Mühling *et al*., 2008) and 1512uR (5′-ACGGHTACCTTGTTACGACTT-3′; Weisburg *et al*., 1991). The PCR reaction was carried out in a 50 μl volume and contained 1.5 mmol l^−1^ MgCl_2_, 200 μmol l^−1^ dNTPs, 1 U of *Taq* DNA polymerase (Invitrogen) and 500 nmol l^−1^ of each of the primers. Template DNA was prepared by incubating a 50 μl aqueous suspension of cells of the bacterial isolates at 98°C for 15 min followed by centrifugation (5 min at 12 000 *g*) to remove fragments of the lysed cells. In general, 1 μl of the supernatant was added to the PCR mix.

The cycle protocol included an initial denaturation step of 4 min at 96°C, followed by 30 cycles (94°C for 60 s, 52°C for 60 s, 72°C for 45 s) and a final extension step at 72°C for 10 min. All PCRs yielded only specific products (i.e. single bands) as judged by electrophoresis of the PCR products on agarose gels. PCR products were purified using ExoSapIT (Amersham Biosciences, Little Chalfont, UK) according to the manufacturer's instructions and used directly for sequence analysis. Nucleotide sequencing of the 3′-terminal end of the 16S rRNA gene fragments was performed using the BigDye Terminator v3.1 cycle sequencing kit (ABI). The primer used in the sequencing reaction was either primer 907F (5′-AAACTCAAAKGAATTGACGG-3′; a modified version of primer 907R of Muyzer *et al*., 1995) or primer Bac1055 (5′-ATGGCTGTCGTCAGCTCGT-3′, a modified version of primer Eco1060 of Lee *et al*., 1993). Sequences were analysed on an ABI 3100 automatic sequencer.

Generally, only one strand of the DNA fragments was sequenced. This proved to be sufficient for the taxonomic identification of the cloned 16S rRNA gene fragments to the genus level using the blast search function within the NCBI database. A summary of the taxonomic composition among the culture collection of isolates screened in this study is available online as supporting information (Table S1).

### Selection of enzymes for screening of activity

A total of 34 enzymes of primary metabolism were screened for activity in 374 isolates. In each case the enzyme activities were assayed in a cell-free extract prepared from bacterial biomass grown to mid-log phase in marine broth and recovered by centrifugation (8000 r.p.m. × 10 min). Harvested cells were disrupted by incubation (60 min at 20°C) with ‘lysomix buffer’ (50 mM phosphate buffer, pH 7.0 containing 5 mg ml^−1^ each of polyethylenimine and lysozyme), and a clear supernatant for assay prepared using a bench microfuge (max. speed for 5 min). The enzymes selected for screening are summarized in Table 1 and details of the biochemical assays used for the screening are provided as supplementary online information (Text S1): in each case where whole-cell preparations were used, the sensitivity of the assays was enhanced by disrupting the cell wall and peripheral plasma membrane of harvested cells using the SembaSonic™ Master Mix (Semba Biosciences, Madison, USA). The selected assays represent 14 different types of enzyme activities: BVMOs, carboxyesterases (including lactone hydrolases), dehalogenases, EC 1.1-type oxidoreductases, EC 1.3-type oxidoreductases, dioxygenases, epoxide hydrolases, laccases, monooxygenases (other than BVMOs), nitrilases, nitrile hydratases, peroxidases, phosphodiesterases, phosphomonoesterases. The enzymes have been broadly categorized (Table 1) as representatives either of core intermediary primary metabolism (‘core’) or alternatively of an activity that might not be found in every bacterium but which might indicate a specialism of that particular bacterium (‘specialist’). An example of a specialist enzyme would be a dehalogenase, which would not be expected to occur in all heterotrophic bacteria; in contrast, most heterotrophic bacteria would be expected to express EC1.1-type dehydrogenases, which are core enzymes of intermediary primary metabolism and widely distributed. In the cases of enzymes such as BVMOs that can serve roles in either primary (Cripps *et al*., 1978) or secondary metabolism (Gibson *et al*., 2005), the nature of the substrates chosen for the screens would favour the selection of those strains expressing catabolic enzymes of primary metabolism.

The enzymes selected for assay had to fulfil five principals. (i) All enzymes had to have established or perceived commercialization potential. That is, either they must catalyse reactions that are difficult or impossible to undertake by conventional chemical catalysis, and/or they undertake enantioselective reactions to generate products with potentially valuable chiral properties (Kirk *et al*., 2002; Bommarius and Riebel-Bommarius, 2004; Faber, 2004). (ii) Liquid phase assays were based on authenticated robust semi-quantitative protocols proven to be suitable for whole-cell preparations of microorganisms (Grogan, 2009; Whittall and Sutton, 2009). (iii) All assays were colourimetric, based either on the use of chromogenic substrates, or on the use of chromogenic development reagents to visualize otherwise colourless product(s) (Reymond, 2005). (iv) Where feasible, liquid phase assays were miniaturized to perform in 96-well microtitre plates, since use of multi-well screening techniques is acknowledged to be faster than traditional agar plate or test tube-based methods (Janes *et al*., 1998). (v) All enzyme activities were assumed to represent the constitutive level of expression of primary metabolic enzymes, because whole-cell preparations were harvested at approximately mid-log phase of growth on unsupplemented marine broth. In addition, various monooxygenase and dioxygenase activities were monitored periodically using solid-phase assays throughout 21 days of growth on marine agar plates that were supplemented with one or more appropriate established enzyme-specific exogenous inducer(s). This means that the detected enzyme activity could have been at an elevated level – in excess of any endogenous level of expression.

### Cluster analysis

Cluster analyses (Clarke and Warwick, 2001) were used to explore the structure of the data set of isolates (identity) and their enzymatic activities utilizing the Primer v6.0 software package (Primer-E, Plymouth, UK). Samples that show the same state in all of the variables (i.e. enzymes that occurred in all isolates) do not have any information of relevance for cluster analyses. Therefore, two different sets of data were used in the analyses. Two enzymes that were present in all isolates (C4- and C16-carboxy esterase) were either included or excluded from the analysis; both analyses resulted in the same cladograms. Results shown are for analyses that included the data on the C4- and C16-carboxy esterase activity.

The data were interrogated in two ways. First, to explore any correlation that might exist between the ability of isolates of specific taxa (at various taxonomic levels: genus, class, phylum) to utilize the various substrates. Second, a hierarchical cluster analysis was utilized to reveal potential patterns in the occurrence of the enzymatic activities among the various isolates.

Cluster analyses were performed on transformed presence/absence data (Table S2) using the simple-matching coefficient (Sokal and Michener, 1958). This is the proportion of characters that have the same state (both negative and positive) in a pair of isolates or enzyme activities to be compared (Sokal and Michener, 1958). The resulting matrices of similarities were clustered by hierarchical agglomerative clustering, with group-average linkage. The significance of divisions within the resulting dendrogram was tested (at *P* = 0.05) using the Similarity Profiles (SIMPROF) test for multivariate structure (Clarke *et al*., 2008).

## Supporting Information

**Text S1.** Details on enzyme screens. **Table S1.** Summary of the bacterial diversity of the 374 marine isolates tested in this study. Identification was based on sequence comparison of a fragment of the 16S rRNA gene with the non-redundant nucleotide (nr/nt) database of the National Center for Bioinformatic Information (NCBI) using the Basic Local Alignment Search Tool (blast: blastn). The closest hits to known genera are given, independent of the level of sequence identity; the exception is one group of isolates that have close similarity to the 16S rRNA gene sequence of Arctic sea ice bacterium ARK10115 (Accession No. AF468367), but no meaningful similarity to any known genus that would allow prediction of their taxonomic identity. Table S2. Summary of 34 enzymatic activities tested in the 374 marine isolates. The results are depicted as binary data whereby ‘1’ and ‘0’ code for presence and absence respectively.
