# Supplementary material for: The biodiscovery potential of marine bacteria: an investigation of phylogeny and function
Source: Microb Biotechnol. 2013 Apr 4;6(4):361–70. doi: 10.1111/1751-7915.12054 (PMC3917471; doi:10.1111/1751-7915.12054)
Supplement: Supplementary file 1 — Text S1. Details on enzyme screens. Table S1. Summary of the bacterial diversity of the 374 marine isolates tested in this study. Identification was based on sequence comparison of a fragment of the 16S rRNA gene with the non-redundant nucleotide (nr/nt) database of the National Center for Bioinformatic Information (NCBI) using the Basic Local Alignment Search Tool (blast: blastn). The closest hits to known genera are given, independent of the level of sequence identity; the exception is one group of isolates that have close similarity to the 16S rRNA gene sequence of Arctic sea ice bacterium ARK10115 (Accession No. AF468367), but no meaningful similarity to any known genus that would allow prediction of their taxonomic identity. Table S2. Summary of 34 enzymatic activities tested in the 374 marine isolates. The results are depicted as binary data whereby ‘1’ and ‘0’ code for presence and absence respectively. [file mbt20006-0361-sd1.zip › mbt2_12054_tableS1.docx]

SI Table S1

Summary of the bacterial diversity of the 374 marine isolates tested in this study. Identification was based on sequence comparison of a fragment of the 16S rRNA gene with the non-redundant nucleotide (nr/nt) database of the National Center for Bioinformatic Information (NCBI) using the Basic Local Alignment Search Tool (BLAST: blastn). The closest hits to known genera are given, independent of the level of sequence identity; the exception is one group of isolates that have close similarity to the 16S rRNA gene sequence of Arctic sea ice bacterium ARK10115 (accession number: AF468367), but no meaningful similarity to any known genus that would allow prediction of their taxonomic identity.

| **Phylum** | **Class** | **Genus** | **No. of isolates** |
| --- | --- | --- | --- |
|  |  |  |  |
| *Actinobacteria* | *Actinobacteria* | *Aeromicrobium* | 3 |
| *Actinobacteria* | *Actinobacteria* | *Agreia* | 1 |
| *Actinobacteria* | *Actinobacteria* | *Arthrobacter* | 1 |
| *Actinobacteria* | *Actinobacteria* | *Cellulomonas* | 1 |
| *Actinobacteria* | *Actinobacteria* | *Dietzia* | 1 |
| *Actinobacteria* | *Actinobacteria* | *Micrococcus* | 3 |
| *Actinobacteria* | *Actinobacteria* | *Nocardioides* | 2 |
| *Actinobacteria* | *Actinobacteria* | *Oerskovia* | 1 |
| *Actinobacteria* | *Actinobacteria* | *Pseudonocardia* | 5 |
| *Actinobacteria* | *Actinobacteria* | *Rhodococcus* | 8 |
| *Actinobacteria* | *Actinobacteria* | *Salinibacterium* | 1 |
| *Actinobacteria* | *Actinobacteria* | *Streptomyces* | 5 |
| *Actinobacteria* | *Actinobacteria* | *Leifsonia* | 1 |
|  |  |  |  |
| *Bacteroidetes* | *Sphingobacteria* | *Algoriphagus* | 1 |
| *Bacteroidetes* | *Flavobacteria* | *Aquimarina* | 2 |
| *Bacteroidetes* | *Flavobacteria* | *Cellulophaga* | 5 |
| *Bacteroidetes* | *Flavobacteria* | *Croceibacter* | 7 |
| *Bacteroidetes* | *Sphingobacteria* | *Cytophaga* | 5 |
| *Bacteroidetes* | *Sphingobacteria* | *Flexibacter* | 2 |
| *Bacteroidetes* | *Sphingobacteria* | *Lacinutrix* | 1 |
| *Bacteroidetes* | *Flavobacteria* | *Maribacter* | 3 |
| *Bacteroidetes* | *Sphingobacteria* | *Marinicola* | 1 |
| *Bacteroidetes* | *Flavobacteria* | *Psychroserpens* | 1 |
| *Bacteroidetes* | *Sphingobacteria* | *Rhodovirga* | 1 |
| *Bacteroidetes* | *Flavobacteria* | *Sufflavibacter* | 1 |
| *Bacteroidetes* | *Flavobacteria* | *Tenacibaculum* | 2 |
| *Bacteroidetes* | *Flavobacteria* | *Zobellia* | 1 |
| *Bacteroidetes* | *Sphingobacteria* | *Balneola* | 1 |
|  |  |  |  |
| *Firmicutes* | *Bacilli* | *Bacillus* | 13 |
| *Firmicutes* | *Bacilli* | *Geobacillus* | 1 |
| *Firmicutes* | *Bacilli* | *Planococcus* | 1 |
|  |  |  |  |
| *Proteobacteria* | *Alphaproteobacteria* | *Agrobacterium* | 1 |
| *Proteobacteria* | *Alphaproteobacteria* | *Sneathiella* | 1 |
| *Proteobacteria* | *Alphaproteobacteria* | *Antarctobacter* | 10 |
| *Proteobacteria* | *Alphaproteobacteria* | (Arctic sea ice bacterium ARK10115) | 12 |
| *Proteobacteria* | *Alphaproteobacteria* | *Brevundimonas* | 3 |
| *Proteobacteria* | *Alphaproteobacteria* | *Erythrobacter* | 4 |
| *Proteobacteria* | *Alphaproteobacteria* | *Leisingera* | 2 |
| *Proteobacteria* | *Alphaproteobacteria* | *Maricaulis* | 1 |
| *Proteobacteria* | *Alphaproteobacteria* | *Octadecabacter* | 1 |
| *Proteobacteria* | *Alphaproteobacteria* | *Pseudorhodobacter* | 4 |
| *Proteobacteria* | *Alphaproteobacteria* | *Rhizobium* | 1 |
| *Proteobacteria* | *Alphaproteobacteria* | *Roseobacter* | 23 |
| *Proteobacteria* | *Alphaproteobacteria* | *Roseovarius* | 4 |
| *Proteobacteria* | *Alphaproteobacteria* | *Ruegeria* | 1 |
| *Proteobacteria* | *Alphaproteobacteria* | *Shimia* | 2 |
| *Proteobacteria* | *Alphaproteobacteria* | *Sphingomonas* | 1 |
| *Proteobacteria* | *Alphaproteobacteria* | *Sphingopyxis* | 2 |
| *Proteobacteria* | *Alphaproteobacteria* | *Sulfitobacter* | 12 |
| *Proteobacteria* | *Alphaproteobacteria* | *Thalassospira* | 2 |
| *Proteobacteria* | *Alphaproteobacteria* | *Vadicella* | 1 |
|  |  |  |  |
| *Proteobacteria* | *Betaproteobacteria* | *Hydrogenophaga* | 1 |
| *Proteobacteria* | *Betaproteobacteria* | *Variovorax* | 1 |
|  |  |  |  |
| *Proteobacteria* | *Gammaproteobacteria* | *Alteromonas* | 6 |
| *Proteobacteria* | *Gammaproteobacteria* | *Colwellia* | 3 |
| *Proteobacteria* | *Gammaproteobacteria* | *Erwinia* | 1 |
| *Proteobacteria* | *Gammaproteobacteria* | *Glaciecola* | 8 |
| *Proteobacteria* | *Gammaproteobacteria* | *Halomonas* | 2 |
| *Proteobacteria* | *Gammaproteobacteria* | *Listonella* | 1 |
| *Proteobacteria* | *Gammaproteobacteria* | *Marinobacter* | 60 |
| *Proteobacteria* | *Gammaproteobacteria* | *Marinobacterium* | 2 |
| *Proteobacteria* | *Gammaproteobacteria* | *Marinomonas* | 19 |
| *Proteobacteria* | *Gammaproteobacteria* | *Melitea* | 1 |
| *Proteobacteria* | *Gammaproteobacteria* | *Oceanospirillum* | 2 |
| *Proteobacteria* | *Gammaproteobacteria* | *Photobacterium* | 1 |
| *Proteobacteria* | *Gammaproteobacteria* | *Pseudoalteromonas* | 22 |
| *Proteobacteria* | *Gammaproteobacteria* | *Pseudomonas* | 31 |
| *Proteobacteria* | *Gammaproteobacteria* | *Psychrobacter* | 1 |
| *Proteobacteria* | *Gammaproteobacteria* | *Psychromonas* | 1 |
| *Proteobacteria* | *Gammaproteobacteria* | *Rheinheimera* | 2 |
| *Proteobacteria* | *Gammaproteobacteria* | *Shewanella* | 23 |
| *Proteobacteria* | *Gammaproteobacteria* | *Spongiibacter* | 2 |
| *Proteobacteria* | *Gammaproteobacteria* | *Stenotrophomonas* | 1 |
| *Proteobacteria* | *Gammaproteobacteria* | *Vibrio* | 11 |
| *Proteobacteria* | *Gammaproteobacteria* | *Oceaniserpentilla* | 1 |
|  |  |  |  |
| *Verrucomicrobia* | *Opitutae* | *Opitutus* | 1 |

SI Table S2

Summary of 34 enzymatic activities tested in the 374 marine isolates. The results are depicted as binary data whereby “1” and “0” code for presence and absence, respectively.
